# Genomic characterisation* of Actinospongicola halichondriae* gen. nov., sp. nov., the first sponge-derived cultivated representative of a new genus within the class *Acidimicrobiia*

**DOI:** 10.1007/s10482-025-02126-4

**Published:** 2025-07-15

**Authors:** Jing Huang, Jutta Wiese, Leon X. Steiner, Tanja Rahn, Erik Borchert, Ute Hentschel

**Affiliations:** 1 GEOMAR Helmholtz Centre for Ocean Research Kiel, RU Marine Ecology, RD3 Marine Symbioses, Wischhofstraße 1-3, 24148 Kiel, Germany; 2 Institute of Microbiology, Chinese Academy of Sciences (IMCAS), Beijing, China; 3 Christian-Albrechts-University (CAU) of Kiel, Kiel, Germany

**Keywords:** *Acidimicrobiia*, Baltic Sea, *Halichondria panicea*, Phosphonate and fucose utilisation, Sponge-association, Stress tolerance

## Abstract

A new strain (named Hal317^T^) of class *Acidimicrobiia* was isolated from the marine breadcrumb sponge *Halichondria panicea* sampled in the Kiel Fjord (Baltic Sea, Germany). Phylogenetic analysis of the 16S rRNA gene sequence revealed similarities from 84.86 to 91.54% to all known type strains of the class *Acidimicrobiia*. Genome comparisons of strain Hal317^T^ with type strains of the family *Iamiaceae* exhibited average nucleotide identities (ANI) < 71.49% and percentage of conserved proteins (POCP) values < 50.30%. Strain Hal317^T^ was determined to be microaerophilic. Optimal growth occurred at 30–35 °C, 1–2% sea salt, and pH 6–7. Major fatty acids identified were C_18:1_ *ω*7*c*, C_16:1_ *ω*7*c*, C_17:1_ *ω*8*c*, and C_16:0_. The major respiratory menaquinone was MK-9(H_8_). The G + C content of strain Hal317^T^ was 68.70%. Based on the polyphasic approach of bacterial classification, strain Hal317^T^ could be distinguished from known genera of the family *Iamiaceae*, and we propose the name *Actinospongicola halichondriae* gen. nov., sp. nov. (= DSM 114536^T^ = LMG 32795^T^). Genomic features indicated that strain Hal317^T^ was characteristic of a sponge-associated lifestyle, such as the potential to metabolise diverse sponge-derived carbohydrates (e.g. chitin, l-fucose) and sulfur compounds (e.g. taurine). Moreover, the genome of strain Hal317^T^ contained stress response-related genes, including those involved in ectoine synthesis and toxin-antitoxin systems. Within the family *Iamiaceae* strain Hal317^T^ revealed unique genomic features, including a non-phosphorylated l-fucose pathway. Our study sheds light on the *Acidimicrobiia*, a class typified by few cultured representatives. Additionally, the new sponge-derived isolate offers valuable insights into the host-bacteria association.

**Supplementary Information:**

The online version contains supplementary material available at 10.1007/s10482-025-02126-4.

## Introduction

In 1996, the first type strain of the class *Acidimicrobiia*, *Acidimicrobium ferrooxidans* DSM 10331^T^ was isolated from copper mine tailings (Clark and Norris 1996). Further clarification of the taxonomic position was implemented by the proposal of the subclass *Acidimicrobidae* (Stackebrandt et al. 1997). In 2012, the subclass was elevated to class and given a new name “*Acidimicrobiia*” (Ludwig et al. 2012). According to LPSN (List of Prokaryotic names with Standing in Nomenclature) the currently valid published taxonomic unit of the class *Acidimicrobiia* includes the heterotypic synonym orders *Acidimicrobiales* and *Iamiales* (Parte et al. 2020), the three families *Acidimicrobiaceae*, *Iamiaceae,* and *Ilumatobacteraceae*, and 16 species. In addition, “*Candidatus* Microthrix parvicella” and “*Candidatus* Microthrix calida” isolated from industrial activated sludge are affiliated with *Microthrix* group of the class *Acidimicrobiia* (Blackall et al. 1996; Levantesi et al. 2006; Hu et al. 2018), which has also been proposed as a provisional family, *Microtrichaceae*, closely related to the family *Ilumatobacteraceae* (Asem et al. 2018).

Species of the family *Acidimicrobiaceae* are often isolated from extreme environments, such as acid mine drainage or geothermal vents. Most of the strains in the family *Acidimicrobiaceae* exhibit acidophilic characteristics and the ability to oxidize or reduce iron (Clark and Norris 1996, Johanson et al. 2009, Itoh et al. 2011, Jones and Johnson 2015, Gonzalez et al. 2020). In contrast, the members of the family *Iamiaceae* and *Ilumatobacteraceae* all grow in neutral or weak alkaline pH environments. Except for the human skin derived *Dermatobacter hominis* (Jo et al. 2023), all other species in family *Iamiaceae* were isolated from aquatic environments. *Aquihabitans daechungensis* was isolated from wastewater sludge (Jin et al. 2013), *Actinomarinicola tropica* was isolated from sediment of South China Sea (He et al. 2020), and *Iamia majanohamensis* from the epidermis of sea cucumber (Kurahashi et al. 2009), *Rhabdothermincola salaria* from saline lake sediment (Gao et al. 2022) and *Rhabdothermincola sediminis* from hot spring sediment (Liu et al. 2019). For the family *Ilumatobacteraceae*, three species of *Ilumatobacter* were found in coastal sandy soils and estuarine sediments, while *Desertimonas flava* was found in desert (Matsumoto et al. 2009, 2013; Asem et al. 2018). Chemolithoautotrophic growth is shown for some members of the class *Acidimicrobiia*. In addition to Fe^2^⁺ oxidation, also sulfur compound oxidation, and probably hydrogen oxidation via hydrogenases as energy sources supports autotrophic growth (Itoh et al. 2011, Gao et al. 2022). Chemolithoautotrophic carbon fixation pathways were reported for the class comprising Calvin-Benson-Bassham (CBB) cycle (Norris et al. 2011; Bay et al. 2021), and reversed oxidative TCA (roTCA) cycle (Gao et al. 2022).

Although only 16 strains of the class *Acidimicrobiia* have been isolated and validly described as new species, recent studies using culture-independent methods have revealed their wide distribution in freshwater (Neuenschwander et al. 2018), coastal (Silva-Solar et al. 2024) and deep-sea hydrothermal sediments (Huang et al. 2023). *Acidimicrobiia* were often enriched and consistently present in different sponge hosts. Sponge-specific *Acidimicrobiales* 16S rRNA gene sequences were identified in sponges *Sarcotragus spinosulus* and *Ircinia variabilis* from the Northeast Atlantic (Hardoim et al. 2012; Hardoim and Costa 2014). Busch et al. (2022) previously identified operational taxonomic units annotated as Actinobacteriota–Acidimicrobiia–Microtrichales–Microtrichaceae–Sva0996_marine_group as core members of the microbial community in deep-sea sponges, due to their occurrence in more than 70% of all sponge samples. Nguyen et al. (2023) reconstructed and characterised 22 metagenome-assembled genomes (MAGs) of *Acidimicrobiia* from sponge, and classified them into six novel species. Recently, the separation between sponge-associated and free-living acidimicrobiial UBA5794 groups in phylogeny and functional potential was indicated (Huang et al. 2025). Despite widely present in sponges, no successful isolation of sponge-derived *Acidimicrobiia* strains have been reported.

In this study, a novel *Acidimicrobiia* strain Hal317^T^ was isolated from Baltic Sea sponge *Halichondria panicea*. A comprehensive analysis of strain Hal317^T^ was depicted in terms of taxonomic classification, genomic content, comparative genome, and physiological characterisation.

## Materials and methods

### Bacterial isolation and cultivation

Strain Hal317^T^ was isolated in the frame of a study on the bacterial diversity of the marine breadcrumb sponge *Halichondria panicea*. Sponge specimens were collected on February 4th, 2019 from Kiel, Schilksee (Baltic Sea, Germany, coordinates: latitude 54.424705, longitude 10.175133) with snorkeling. Samples were transferred in Kautex plastic bottles to the laboratory within 2 h after collection. A piece of the sponge (10.8 g) was washed three times with 0.2 µm filtrated and autoclaved Baltic Sea water (BSW) to remove loosely attached microorganisms. Homogenisation of the sponge specimen was performed with 30 ml BSW in a 50 ml Falcon plastic tube using an Ultraturrax (30 s at 17,500 rpm). Dilutions were prepared with BSW from 10^–1^ to 10^–4^. 100 µl of the dilutions were transferred onto Baltic Sea medium (1 g *N*-acetyl-d-mannosamine, 15 g Bacto-Agar, 1000 ml BSW, pH 7.5). The media plates were incubated at 25 °C under standard atmospheric conditions. *N*-acetyl-d-mannosamine was selected as supplement of the medium, because it is structurally similar to *N*-acetyl-d-glucosamine, the monomer of chitin, which can be utilised by some sponge-associated bacteria (Kohn et al. 2020; Meunier et al. 2024). This medium was used for our isolation campaigns in 2018/2019, originally with the aim to isolate *Candidatus* Halichondribacter symbioticus, an uncultured member of the family *Paracoccaceae* (former: *Rhodobacteraceae*), from the sponge *H. panicea*. From genomic features we assumed that media based on Baltic Sea water and supplemented with *N*-acetyl-d-glucosamine, *N*-acetyl-mannosamine, or taurine might support the growth of *Candidatus* Halichondribacter symbioticus. Of the different media used for the isolation approaches only agar plates supplemented with *N*-acetyl-d-mannosamine supported growth of colonies appearing as “transparent grease” that were observed after incubation of 6 weeks. Since this colony type was not observed in any of our former isolation experiments it attracted our attention and a sub-cultivation of strain Hal317^T^ was carried out. The sub-cultivation was performed onto Marine Agar Difco 2216 (Becton Dickinson and Company, New Jersey, USA) for 7 days at 25 °C. Cell material of strain Hal317^T^ was cryopreserved at − 80 °C using the Cryobank System (Mast Diagnostica GmbH, Reinfeld, Germany). Strain Hal317^T^ was deposited in the public culture collections BCCM/LMG and DSMZ with the deposition numbers LMG 32795^T^ and DSM 114536^T^, respectively.

### 16S rRNA gene sequencing and phylogenetic analysis

Genomic DNA of strain Hal317^T^ was extracted using the DNeasy Blood & Tissue Kit (Qiagen GmbH, Hilden, Germany) for Gram-positive bacteria according to the manufacturer’s instructions. Amplification of the 16S rRNA gene sequence was performed with the primers Eub27F (5′-GAGTTTGAT CCTGGCTCAG-3′) and Univ1492R (5′-GGTTACCTTGTTACGACTT-3′). Sequencing was carried out with Sanger sequencing at Eurofins Genomics (Ebersberg, Germany) using the primers 534R (5′-ATTACCGCGGCTGCTGG-3′), 342 F (5′-TACGGGAGGCAGCAG-3′), and Univ1492R. The sequenced DNA fragments were assembled to a partial 16S rRNA gene sequence and its quality was assessed using ChromasPro 2.1. (Technelysium Pty Ltd, Brisbane, Australia). The partial 16S rRNA gene sequence was deposited under the Genbank accession number MT406698. PCR-based 16S rRNA gene sequence (1493 bp) is identical with the full-length sequence derived from the genome (1517 bp) in the overlapping region. 16S rRNA gene sequences used for phylogenetic analyses were collected from EzBioCloud 16S database applying the feature “16S_based ID” (Chalita et al. 2024). The full-length genome-derived 16S rRNA gene sequence of strain Hal317^T^ was aligned to the 16 type strains known for the class *Acidimicrobiia* and to *Streptomyces poriferorum* P01-B04^T^ as the outgroup by the ClustalW feature of MEGA version 11.0.13 (Tamura et al. 2021). Phylogenetic trees were calculated using three methods to ensure the consistency of the tree topology: (i) Neighbor-Joining (NJ) (Saitou and Nei 1987) by computing the evolutionary distances with the Maximum Composite Likelihood method (Tamura et al. 2004), (ii) Minimum Evolution (ME) combined with the Maximum Composite Likelihood method and Close-Neighbor-Interchange (CNI) algorithm (RzHetsky and Nei 1992), and (iii) Maximum-Likelihood (ML) together with the General Time Reversible model (Tamura and Nei 1993). NJ, ME, and ML phylogenetic trees were calculated using MEGA version 11.0.13 with 1000 bootstrap replications. Trees were drawn to scale and included branch lengths measured in the units of the number of base substitutions per site.

### Whole-genome sequencing

Strain Hal317^T^ was cultivated in 100 ml half-concentrated MB medium at 28 °C for 7 days and 120 rpm. Cell suspension was centrifuged and the cell pellet was frozen at − 20 °C before DNA-extraction. *A. daechungensis* DSM 27986^ T^ was grown on 10 R_2_A medium plates (0.5 g yeast, 0.5 g proteose peptone, 0.5 g casamino acids, 0.5 g d-glucose monohydrate, 0.5 g starch, 0.3 g Na-pyruvate, 0.3 g K_2_HPO_4_, 0.3 MgSO_4_·7H_2_O, 15 g Bacto-agar, 1000 ml deionised water) at 28 °C for 14 days. DNA from cell material of both strains was extracted with Qiagen Genomic-tip 100/G (Hilden, Germany) according to the standard protocol for bacteria by the manufacturer. Quality of DNA extract was assessed with NanoDrop (Thermo Fisher Scientific, Germany) measurements. Extracted DNA of strain Hal317^T^ revealed a concentration of 284 ng/µl, an A260/280 ratio of 1.88, and an A260/230 ratio of 2.08. Concentration of DNA of *A. daechungensis* DSM 27986^T^ was 474 ng/µl, A260/280 ratio was 1.92, and A260/230 ratio was 2.03.

Genomes were sequenced with Oxford Nanopore Technology (ONT) at Eurofins Genomics (Ebersberg, Germany). Raw nanopore sequencing reads were assessed for quality and filtering. Filtlong v0.2.1 (https://github.com/rrwick/Filtlong) was used to remove short and low-quality reads from the raw nanopore sequencing data. The high-quality nanopore sequencing reads were then used for de novo assembly of the bacterial genome. The assembly is performed using Flye v2.9.3 (Kolmogorov et al. 2019) with parameters optimized for bacterial genomes. The resulting contigs are further polished using Medaka v1.8 (https://github.com/nanoporetech/medaka) to improve base accuracy.

Annotation was carried out with eggNOG-mapper (http://eggnog-mapper.embl.de/) (Cantalapiedra et al. 2021), AntiSMASH (Blin et al. 2023), PGAP (Tatusova et al. 2016), METABOLIC (Zhou et al. 2022), and Bakta v1.7.0 (Schwengers et al. 2021). General genomic features were calculated using CheckM (Parks et al. 2015) and Prokka 1.3 (Seemann 2014). Average amino acid identity (AAI) and average nucleotide identities (ANI) were determined with compareM tool (https://github.com/dparks1134/CompareM) and the ANI calculator provided by EzBioCloud (Yoon et al. 2017), respectly. Percentage of conserved proteins (POCP) was calculated for genus delineation using POCP-nf v2.3.0 (Qin et al. 2014; Hölzer 2024). Digital DNA-DNA hybridisation (dDDH) values were computed using the dDDH calculator from the Type (Strain) Genome Server (TYGS) (Meier-Kolthoff and Göker 2019). Genome-based phylogeny was calculated with a concatenated alignment of 120 conserved bacterial marker proteins, predicted by GTDB-Tk from strain Hal317^T^ and publicly available acidimicrobial type strain genomes, applying the NJ-, ME-, and ML-methods in MEGA version 11.0.13.

### Morphology

Morphological characteristics of strain Hal317^T^ were analysed after cultivation in 100 ml half-concentrated MB medium at 28 °C for 7 days and 120 rpm. Colony morphology was observed using a loupe, while cell morphology and motility were examined using light microscopy (Carl Zeiss Axiophot epifluorescence microscope). Gram-staining was carried out with the bioMérieux Color Gram 2 Test Kit (bioMérieux Deutschland GmbH, Nürtingen, Germany) according to the instructions for use.

### Physiology and chemotaxonom

Salinity-dependent growth was analysed on a medium (5.0 g BD Bacto™ Peptone, 1.0 g BD Bacto™ Yeast Extract, 15.0 g BD Bacto™ Agar, 1 L of deionised water) supplemented with 0–7% (w/v) NaCl and 0–7% (w/v) Tropic Marine sea salt classic (Wartenberg, Germany) respectively, in 1% intervals. The cultures were incubated at 25 °C for 7 days. The effect of temperature-dependent on the growth was assessed at 5–40 °C (intervals of 5 °C) on MB for 7 days. pH-dependent growth of the strain was assessed on MB at 25 °C for 7 days at pH levels 5.0, 6.0, 6.5, 7.5, 8.0, 8.5, 9.0, and 9.5, adjusted using 1 M NaOH or 1 M HCl solutions. Optimum values were confirmed by the strongest growth of strain Hal317^T^. Oxygen requirement was determined using the aerobic/anaerobic test tube method (Hogg 2013) on soft agar MB medium (7.48 g Difco™ Marine Broth 2216, 1.2 g BD Bacto™ Agar in 200 ml of deionised water) and incubation at 25 °C for 7 days. 3% (v/v) hydrogen peroxide was added to colonies of the strains and the formation of gas bubbles (Iwase et al. 2013) was observed to determine catalase activity. Oxidase activity was tested by smearing colonies onto a filter paper disc soaked with bioMérieux oxidase reagent (*N*,*N*,*N*,*N*-tetramethyl-1,4-phenylenediamine) and observing the development of a violet to purple coloration within 1030 s according to the manufacturer’s instructions.

Enzymatic activities were determined using the semi-quantitative API® ZYM test kit (bioMérieux) as indicated by the manufacturer. Cell material of strain Hal317^T^ was suspended in 0.9% NaCl solution and used as inoculum. Incubation was performed for 18 h at 25 °C.

Chemotaxonomic characteristics (cellular fatty acids, respiratory quinones, polar lipids, whole-cell sugars, and diaminopimelic acid isomers) of strain Hal317^T^ were analysed by DSMZ Services (Leibniz Institute DSMZ, Braunschweig, Germany) using the cell suspension of a 7-day old culture grown in half-concentrated MB medium at 28 °C and 120 rpm. In brief, cellular fatty acids were analysed after conversion into fatty acid methyl esters (FAMEs) by saponification, methylation and extraction following the protocol of Sasser (Sasser 1990). The fatty acid methyl esters mixtures were separated by gas chromatography and detected by a flame ionization detector. In subsequent analysis, fatty acids were identified by a GC–MS run, on an Agilent GC–MS 7000D system (Vieira et al. 2021). Peaks were identified based on retention time and mass spectra. Respiratory quinones were extracted from fresh cell material using hexane and were further purified by a silica-based solid phase extraction. Purified samples were analysed by HPLC using a reverse phase column recording absorption spectra (Vieira et al. 2021). 270 nm for ubiquinones and 326 nm for menaquinones were used for a relative quantification. For complex mixtures, samples are further analysed on an UHPLC-ESI-qTOF system. Polar lipids are extracted from freeze dried cell material using a chloroform: methanol: 0.3% aqueous NaCl mixture, polar lipids are recovered into the chloroform phase (modified after Bligh and Dyer 1959). Polar lipids are separated by two-dimensional silica gel thin layer chromatography. The first direction is developed in chloroform: methanol: water, and the second in chloroform: methanol: acetic acid: water. Total lipid material is detected using molybdatophosphoric acid and specific functional groups detected using spray reagents specific for defined functional groups (Tindall et al. 2007). Diagnostic sugars in whole-cell hydrolysates (1N H_2_SO_4_, 100 °C, 2 h) were analysed by thin layer chromatography (TLC) on cellulose plates (ScHumann 2011). Whole-cell hydrolysates (4N HCl, 100 °C, 16 h) are examined by TLC on cellulose plates for the presence of 2,6-diaminopimelic acid (Dpm) isomers or 2,6-diamino-3-hydroxypimelic acid (OH-Dpm).

## Results and discussion

### 16S rRNA gene and whole-genome phylogeny

**Fig. 1:**
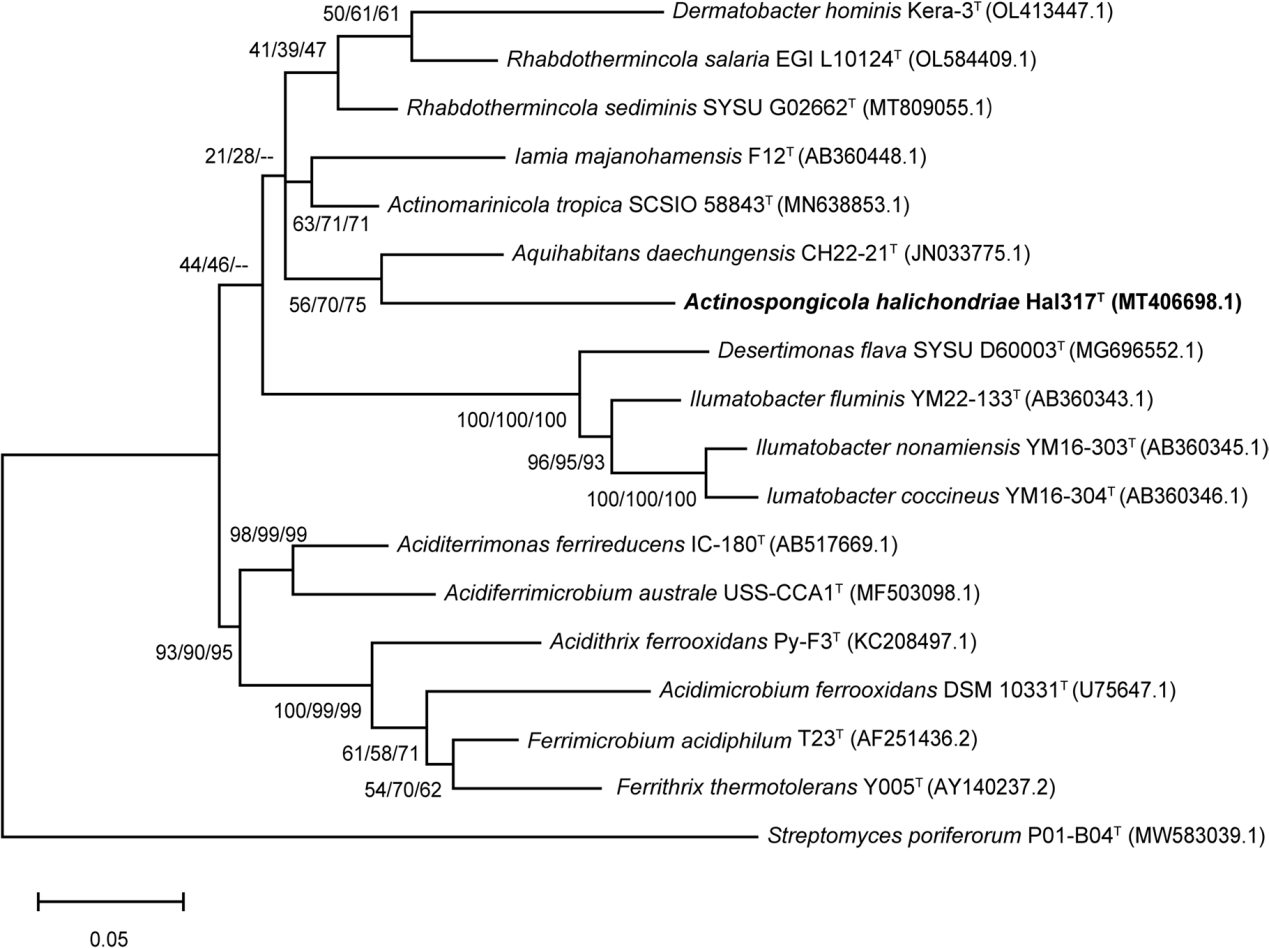
Phylogenetic relationships of Hal317^T^ based on 16S rRNA gene sequences using the Maximum-Likelihood method. Bootstrap values based on 1000 replications are shown next to the branches (ML/NJ/ME). *Streptomyces poriferorum* P01-B04^T^ was used as an outgroup

16S rRNA gene sequence similarity within all type strains of class *Acidimicrobiia* is in the range from 86.74 to 91.54% indicating that strain Hal317^T^ belongs to a new species according to a < 98.65% threshold (Kim et al. 2014). The strain Hal317^T^ clustered with *A. daechungensis* DSM 27986^T^ (90.94%) in all the 16S rRNA phylogenetic tree constructed by NJ, ML and ME methods (Fig. 1) with relatively low bootstrap values (55, 70, and 75, respectively), but it was most similar to *A. tropica* SCSIO 58843^T^ (91.54%), far below the threshold for a new genus (94.5%) (Yarza et al. 2014). In the 16S rRNA phylogenetic tree, the families *Acidimicrobiaceae* and *Ilumatobacteraceae* formed well-supported branches with high bootstrap values. In contrast, the nodes within the family *Iamiaceae* generally showed low support. Additional new type strain sequences from the family *Iamiaceae* may be required to better resolve both the internal relationships within this family and its phylogenetic placement relative to *Acidimicrobiaceae* and *Ilumatobacteraceae*.

**Fig. 2:**
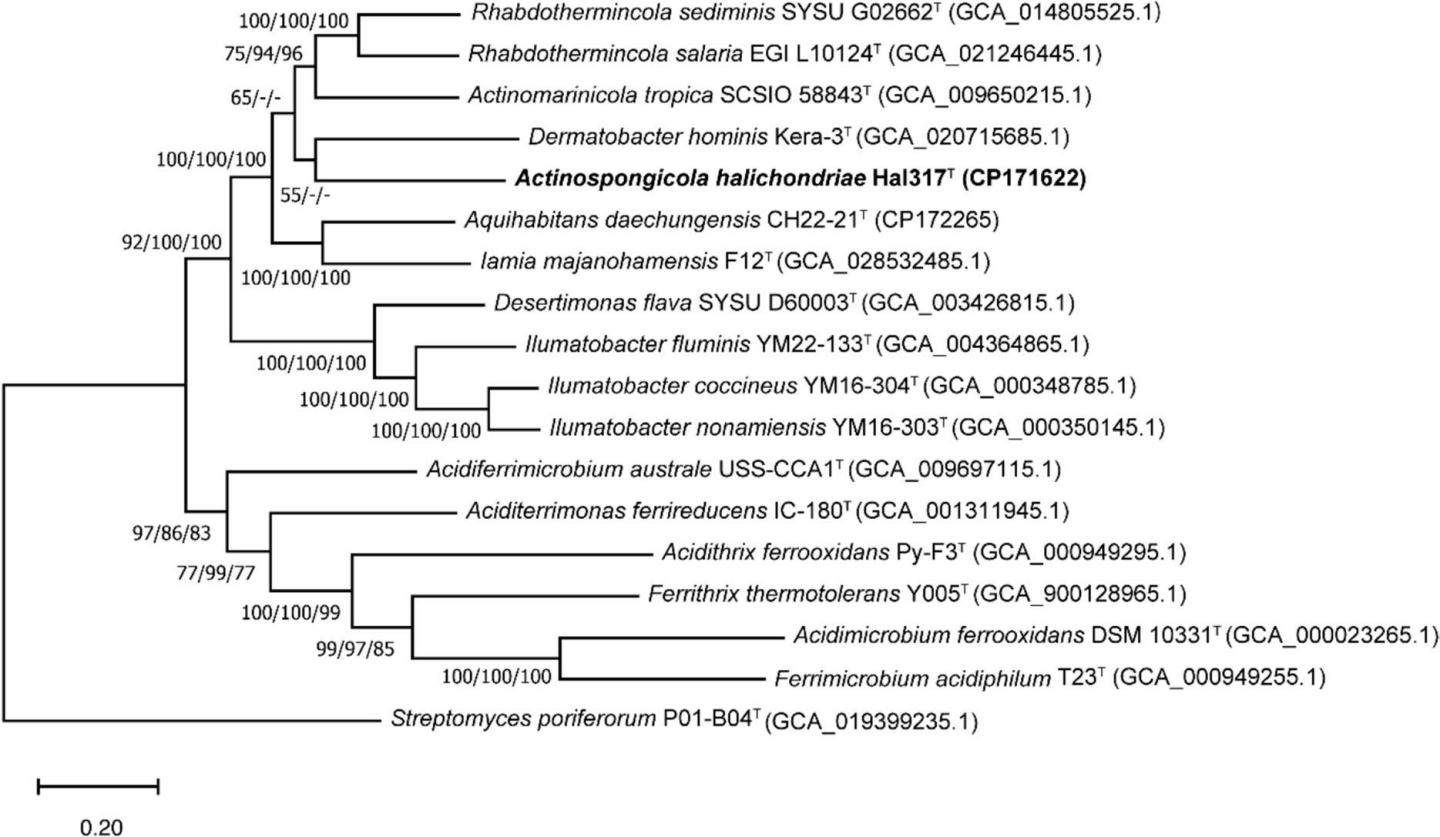
Genomic phylogeny of strain Hal317^T^ was inferred using 120 single copy marker proteins identified by GTDB-Tk pipeline. Bootstrap values based on 1000 replications are shown next to the branches (ML/NJ/ME). *Streptomyces poriferorum* P01-B04^T^ was used as an outgroup

The genome data of type strains from *Acidimicrobiia* were used for phylogenomic analysis. Based on a whole-genome phylogenomic tree constructed using the concatenation of 120 single copy marker proteins with ML method, strain Hal317^T^ clustered with *D. hominis* Kera-3^T^, but at low branch supporting rate (55%) (Fig. 2). In the phylogenomic tree calculated by NJ and ME methods, strain Hal317^T^ was also most close to *D. hominis* Kera-3^T^ but with bootstrap values below 50%.

We could not confidently determine which reference strain is more closely related to strain Hal317^T^ because of the low branch support values. Differences between the 16S rRNA gene sequence phylogeny and the whole-genome based phylogenetic trees could occur, because the 16S rRNA gene trees only infers the phylogenetic relationships from the single conserved gene in contrast to 120 single copy marker proteins used for the phylogenomic tree.

### Genomic characterisation

Strain Hal317^T^ showed the lowest DNA G + C content of 69% (Table 1) among the existing type strains of the family *Iamiaceae*. The mean dDDH values determined for strain Hal317^T^ with type strains were in the range of 13.6–14.2% (Table 2). ANI values between strain Hal317^T^ and the other type strains from the family *Iamiaceae* were < 71.49% (Table 2), way below the threshold of species delineation (95%) (Jain et al. 2018). By analysing the ANI value of hundreds of genera, Barco et al. (2020) reported genus demarcation boundary of ANI with a mean value of 73.98% and a median value of 73.11%, but so far no specific thresholds can be universally employed in bacteria genus demarcation (Barco et al. 2020; Liu et al. 2022). In reference to the mean ANI value reported above, strain Hal317^T^ potentially represents a novel genus. Moreover, the AAI values between strain Hal317^T^ and the other type strains were in the range of 56.53–59.16% (Table 2), lower than the proposed 60% threshold for AAI genus delineation (Riesco and Trujillo 2024). The POCP values between strain Hal317^T^ and other *Iamiaceae* stains ranged from 46.04 to 50.30% (Table 2). Although the pairwise POCP values of Hal317^T^ with *I. majanohamensis* F12^T^ (50.30%) and *R. sediminis* SYSU G02662^T^ (50.04%) were slightly above the 50% genus boundary (Qin et al. 2014; Hölzer 2024), Hal317^T^ should still be considered a novel genus due to the discrepancies in ANI, AAI, and 16S rRNA gene sequence similarity.

**Table 1:** Comparison of the general genomic features of strain Hal317^T^ and the other type strains in the family *Iamiaceae* (this study)

Genome feature	1	2	3	4	5	6	7
Size (Mb)	4.08	3.72	4.16	4.66	4.58	3.91	3.29
Number of contigs (> 1000 bp)	1	1	1	1	1	7	49
N50 (bp)	4,081,508	3,715,454	4,162,068	4,661,060	4,576,919	2,005,197	175,816
G + C content (%)	69	73	71	73	75	72	71
Completeness (%)	97.56	98.84	91.24	95.78	99.44	97.83	100.00
Contamination (%)	1.15	1.29	1.57	0.13	0.67	0.25	0.42
Coding sequences	3986	3658	4087	4288	4304	3579	3199
Coding density	0.938	0.933	0.897	0.918	0.912	0.911	0.924
Pseudogenes	1	0	31	0	1	0	0
tRNA	48	49	47	46	51	47	47
rRNA	3	3	3	3	3	3	3

Strains: 1, Hal317^T^ (CP107030); 2, *Actinomarinicola tropica* SCSIO 58843^T^ (GCA_009650215.1); 3, *Aquihabitans daechungensis* CH22-21^T^ (CP172265); 4, *Dermatobacter hominis* Kera-3^T^ (GCA_020715685.1); 5, *Iamia majanohamensis* F12^T^ (GCA_028532485.1); 6, *Rhabdothermincola salaria* EGI L10124^T^ (GCA_021246445.1); 7, *Rhabdothermincola sediminis* SYSU G02662^T^ (GCA_014805525.1)

**Table 2:** Comparison of ANI, AAI, dDDH and POCP values between strain Hal317^T^ and phylogenetically related type strains in the family *Iamiaceae* (this study)

Index	1 (%)	2 (%)	3 (%)	4 (%)	5 (%)	6 (%)	7 (%)
ANI	100	71.35	71.49	70.99	71.46	70.71	70.58
AAI	100	58.85	58.41	56.53	57.95	58.30	59.16
dDDH (d6)	100	14.20	13.90	13.70	13.80	13.90	13.60
POCP	100	49.12	46.72	46.04	50.30	48.75	50.04

Strains: 1, Hal317^T^ (CP107030); 2, *Actinomarinicola tropica* SCSIO 58843^T^ (GCA_009650215.1); 3, *Aquihabitans daechungensis* CH22-21^T^ (CP172265); 4, *Dermatobacter hominis* Kera-3^T^ (GCA_020715685.1); 5, *Iamia majanohamensis* F12^T^ (GCA_028532485.1); 6, *Rhabdothermincola salaria* EGI L10124^T^ (GCA_021246445.1); 7, *Rhabdothermincola sediminis* SYSU G02662^T^ (GCA_014805525.1)

**Fig. 3:**
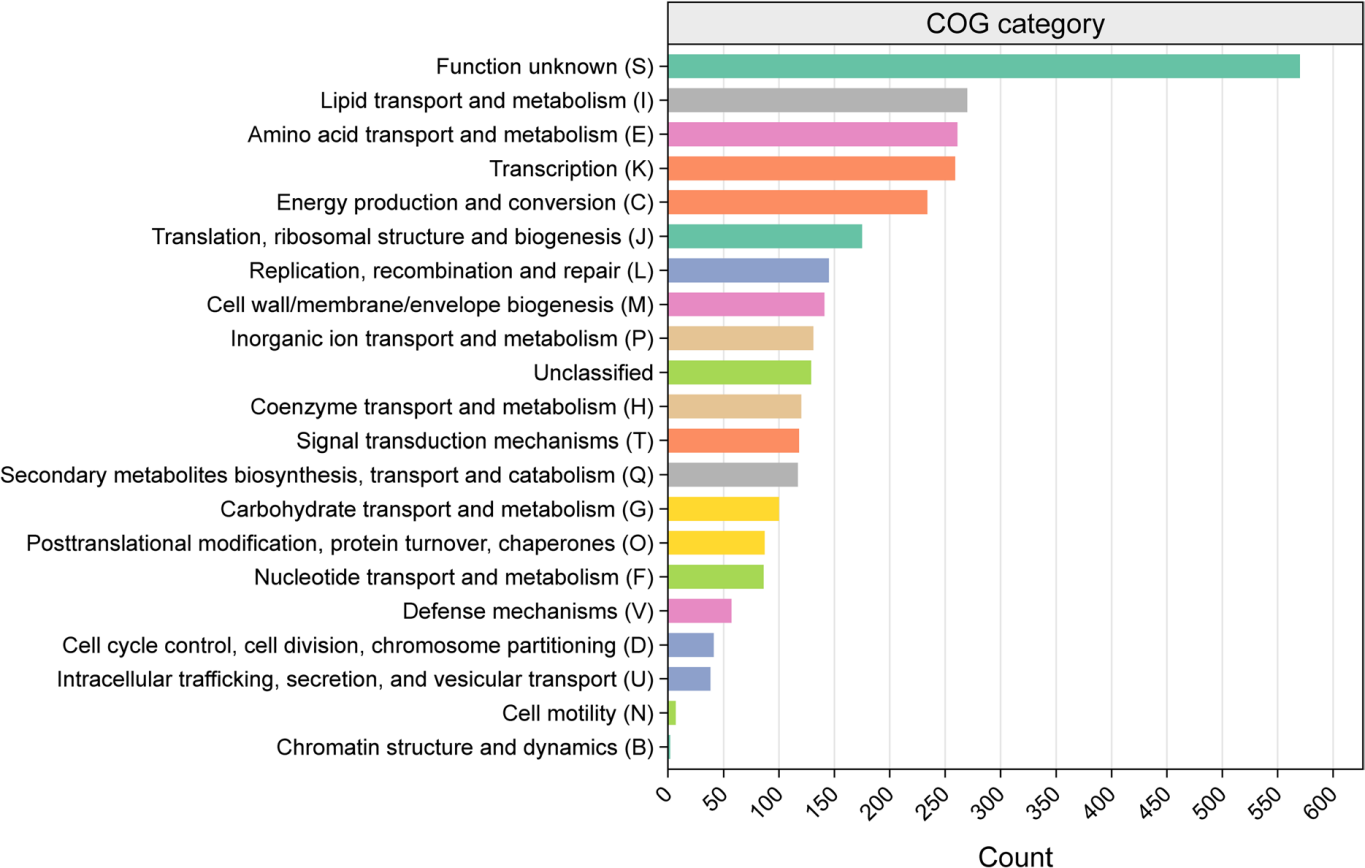
COG profile of the predicted genes in strain Hal317^T^ genome

From the predicted genes, a total of 74.26% (2960/3986) assignments into 21/24 COG functional categories were made, with the majority belonging to function unknown (S), lipid transport and metabolism (I), amino acid transport and metabolism (E), and transcription (K) (Fig. 3). In the genome sequences of strain Hal317^T^, the function unknown (S) was the largest COG category (18.45%), which indicates the functional novelty of genes not defined in the reference database. Notably, the numbers of genes belonging to the lipid transport and metabolism (I) and amino acid transport and metabolism (E) are approximately twice that of carbohydrate transport and metabolism (G), implying that strain Hal317^T^ might prefer metabolising lipids and amino acids than carbohydrates. The diversity of lipids, especially fatty acids, in sponges surpasses that of other marine animals (Rod'kina 2005). The extracellular matrix of sponges is a rich reservoir of proteoglycans, adhesive glycoproteins and collagen-like proteins, (Fernàndez-Busquets and Burger 1999) acting as the carbon and nitrogen source required by microorganisms. It was reported that sponge derived proteins improved the growth of symbiont microbes (De Rosa et al. 2003).

### Genome traits of strain Hal317^T^ indicating a host-associated lifestyle

**Fig. 4:**
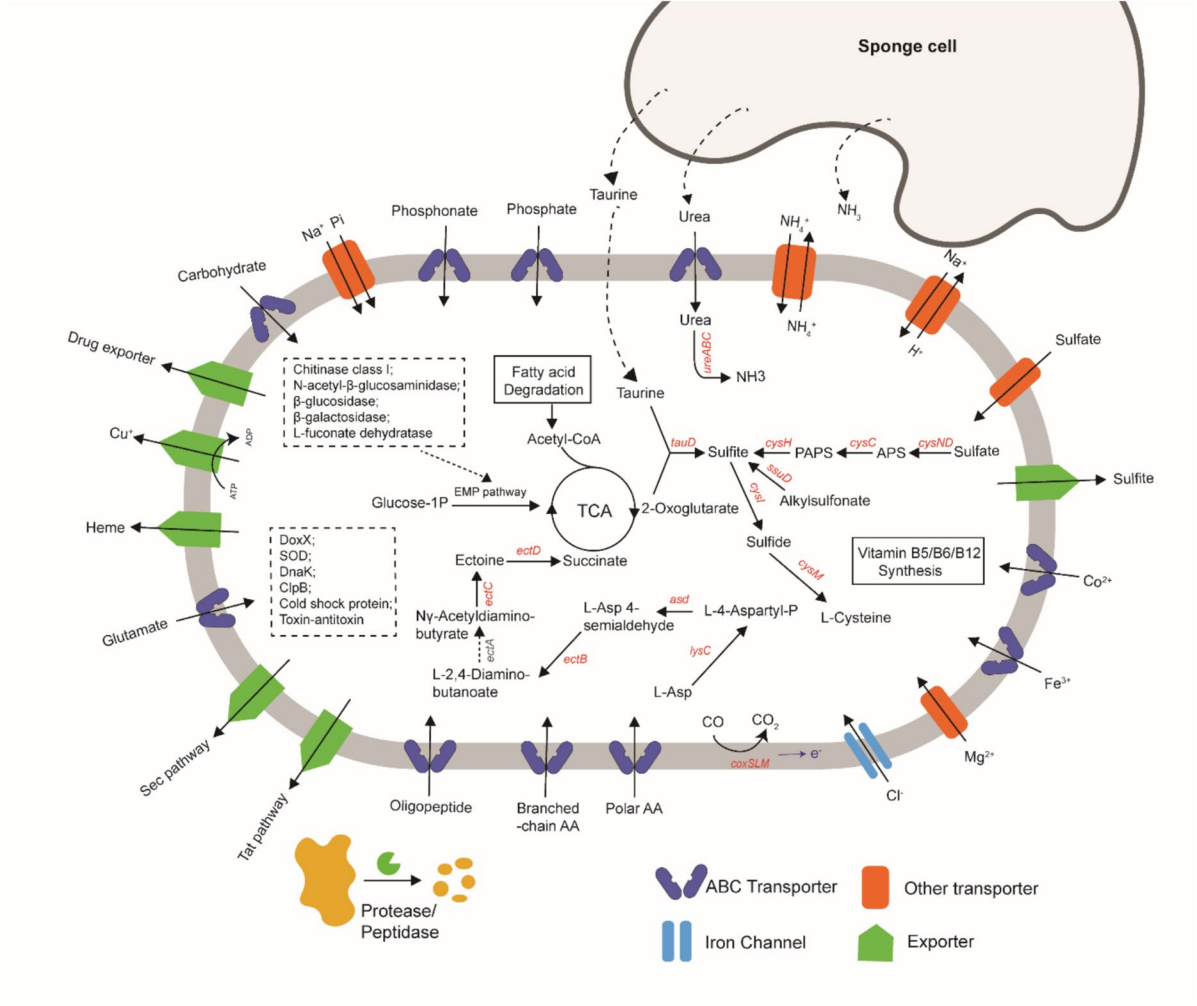
Genome scheme of the strain Hal317^T^. Genes or pathways related to protease, transporter system, carbohydrate utilisation, fatty acid degradation, TCA cycle, urea utilisation, taurine and sulfate metabolism, vitamins B5, B6, and B12 synthesis, environmental stress response system, ectoine synthesis and degradation, and CO oxidation are shown

The strain might be able to feed on a broad spectrum of carbon sources being present in sponge. Carbohydrate-active enzymes (CAZymes) of glycoside hydrolases families such as GH2 (2 genes), GH3 (2 genes), and GH29 (3 genes), as well as ABC transporters of carbohydrates were shown in the genome (Fig. 4). β-galactosidase (EC:3.2.1.23), which belongs to GH2, might be involved in the degradation of galactoside-specific lectins, known to be produced by *Halichondria okadai* (Kawsar et al. 2008), by strain Hal317^T^. GH3 refers to enzymes able to metabolise biomolecules with a β-glucoside linkage such as cellulose and chitin. Genes encoding enzymes of chitinase class I (EC:3.2.1.14) and *N*-acetyl-β-glucosaminidase (EC:3.2.1.52) were revealed by KEGG annotation of Hal317^T^ genome. Chitin plays a significant structural role within the skeletal fibers of sponges belonging to the class *Demospongia* (Wysokowski et al. 2013) and might serve as the natural and rich source of carbohydrate for inhabiting microbes (Raimundo et al. 2021). GH29 family enzymes are known to degrade fucoidan, a complex fucosaccharide prominent in brown algae. Strain Hal317^T^ exhibited genes coding for α-L-fucosidase (EC:3.2.1.51) hydrolysing fucoidan to its monomer α-l-fucose. l-fucose is assumed to be present in high concentrations in the sponge tissue (Sizikov et al. 2020). Furthermore, in Hal317^T^ genome, the non-phosphorylated l-fucose pathway (Watanabe 2024) was present, which could metabolise α-l-fucose to pyruvate and lactate with the contribution of the enzymes d-threo-aldose 1-dehydrogenase (EC:1.1.1.122; l-fucose→l-fucono-1,5-Lactone); l-fucono-1,5-lactonase (EC:3.1.1.120; l-fucono-1,5-lactone→l-fuconate); l-fuconate dehydratase (EC:4.2.1.68; l-fuconate→2-dehydro-3-deoxy-l-fuconate). The same pathway was reported for *Petrosia ficiformis* associated *Verrumicrobia* (Sizikov et al. 2020). By the activity of carbon monoxide dehydrogenase, encoded by genes *coxL*, *coxM*, and *coxS*, strain Hal317^T^ has the potential ability to oxidise carbon monoxide (CO) to carbon dioxide (CO_2_) under aerobic conditions. Just like CO-oxidising members of the sponge bacterial community, genome analysis suggests that Hal317^T^ may be able to generate reductive energy from the oxidation of CO (Thomas et al. 2010; Feng et al. 2019).

Hal317^T^ could get access to amino acids through the activity of proteases and peptidases. Based on the annotation results using MEROPS database, the groups S12 (serine-type D-Ala-D-Ala carboxypeptidases) and S33 (mainly exopeptidases that act at the N-terminus of peptides) are most enriched in the Hal317^T^ genome. In addition, a cell wall-associated protease was found in the genome of strain Hal317^T^ based on the KEGG annotation (Table 3). Exoenzymes such as proteases, peptidases, and chitinases might be transported across the cytoplasmic membrane to the cell envelope or to the extracellular space by translocase proteins, such as the Sec and Tat systems (Fig. 4). The extracellular glutamate could be incorporated into Hal317^T^ via ABC transporter protein GluABC. Glutamate dehydrogenase (GDH) metabolises glutamate to α-ketoglutarate and ammonia. The obtaining of glutamate might provide benefit to the strain as an additional carbon source, entering the citric acid cycle via α-ketoglutarate, as well as serving as a nitrogen source. As shown for the marine sponge *Tethya wilhelma* and for the fresh water sponge *Ephydatia muelleri*, glutamate induces the coordinated contraction of canals of sponges and affects osculum closure and pumping activity (Ellwanger et al. 2007; Elliot and Leys 2010). Due to these observations, the coordinating behavior might be partly affected by the uptake of glutamate through strain Hal317^T^.

**Table 3:** Differential characteristics of strain Hal317^T^ and phylogenetically related type strains in the family *Iamiaceae* derived from the comparative genome analysis (this study)

Cluster	Description	1	2	3	4	5	6	7
K03119	*tauD*; taurine dioxygenase [EC:1.14.11.17]	+	−	+	+	+	+	−
K03791	Putative chitinase	+	−	−	−	−	+	−
K01428	*ureC*; urease subunit alpha [EC:3.5.1.5]	+	−	−	+	−	−	−
K01429	*ureB*; urease subunit beta [EC:3.5.1.5]	+	−	−	+	−	−	−
K01430	*ureA*; urease subunit gamma [EC:3.5.1.5]	+	−	−	+	−	−	−
K00836	*ectB*, *dat*; diaminobutyrate-2-oxoglutarate transaminase [EC:2.6.1.76]	+	+	−	−	+	+	−
K06720	*ectC*; L-ectoine synthase [EC:4.2.1.108]	+	+	−	−	+	+	−
K07746	parD1_3_4; antitoxin ParD1/3/4	+	−	−	+	−	+	−
K19092	parE1_3_4; antitoxin ParE1/3/4	+	−	−	−	−	+	−
K07116	*pvdQ*, *quiP*; acyl-homoserine-lactone acylase [EC:3.5.1.97]	+	−	+	−	−	−	+
K13075	*ahlD*, *aiiA*, *attM*, *blcC*; *N*-acyl homoserine lactone hydrolase [EC:3.1.1.81]	+	−	−	+	−	−	−
K07657	*phoB*; two-component system, OmpR family, phosphate regulon response regulator PhoB	+	−	−	−	−	−	−
K06193	*phnA*; protein PhnA	+	−	−	−	−	−	−
K02041	*phnC*; phosphonate transport system ATP-binding protein [EC:7.3.2.2]	+	−	−	−	−	−	−
K02042	*phnE*; phosphonate transport system permease protein	+	−	−	−	−	−	−
K02044	*phnD*; phosphonate transport system substrate-binding protein	+	−	−	−	−	−	−
K10005	*gluB*; glutamate transport system substrate-binding protein	+	−	−	−	−	−	−
K10006	*gluC*; glutamate transport system permease protein	+	−	−	−	−	−	−
K10007	*gluD*; glutamate transport system permease protein	+	−	−	−	−	−	−
K00064	d-threo-aldose 1-dehydrogenase [EC:1.1.1.122]	+	−	−	−	−	−	−
K18334	*fucD*; l-fuconate dehydratase [EC:4.2.1.68]	+	−	−	−	−	−	−
K07046	l-fucono-1,5-lactonase [EC:3.1.1.120]	+	−	−	−	−	−	−
K13274	*wprA*; cell wall-associated protease [EC:3.4.21.-]	+	−	−	−	−	−	−
K13277	*epr*; minor extracellular protease Epr [EC:3.4.21.-]	+	−	−	−	−	−	−

Strains: 1, Hal317^T^ (CP107030); 2, *Actinomarinicola tropica* SCSIO 58843^T^ (GCA_009650215.1); 3, *Aquihabitans daechungensis* CH22-21^T^ (CP172265); 4, *Dermatobacter hominis* Kera-3^T^ (GCA_020715685.1); 5, *Iamia majanohamensis* F12^T^ (GCA_028532485.1); 6, *Rhabdothermincola salaria* EGI L10124^T^ (GCA_021246445.1); 7, *Rhabdothermincola sediminis* SYSU G02662^T^ (GCA_014805525.1); +, present; -, absent

Like sponge-associated acidimicrobial species (Nguyen et al. 2023), strain Hal317^T^ could acquire sulfur from different compounds such as sulfate, taurine, and alkylsulfonates. Sulfate from the environment could be transferred into the cell of strain Hal317^T^ by a sulfate transporter. The tri-functional sulfate-activating complex (CysDNC) converts sulfate to adenosine 5′-phosphosulfate (APS) and APS to 3′-phosphoadenosine-5′-phosphosulfate (PAPS). PAPS is converted to sulfite by phosphoadenosine phosphosulfate reductase (CysH). Sulfite is also the metabolite of the taurine dioxygenase (TauD) using taurine as substrate and of alkanesulfonate monooxygenase (SsuD) using alkylsulfonates as substrates. Sulfite is metabolised by sulfite reductase (β-subunit CysI) to sulfide, which is used by cysteine synthase (CysM) to synthesise the sulfur containing amino acid cysteine. An exporter transfers excess sulfite out of the cell. Taurine dioxygenase (TauD) present in the genome of Hal 317^T^ could catalyze the following reaction: taurine + α-ketoglutarate (= 2-oxogluturate) + O_2_→sulfite + aminoacetaldehyde + succinate + CO_2_. Taurine is a ubiquitously occurring sulfonate metabolite in marine sponges, oftentimes as one of the most abundant free sulfur-containing amino acids (up to 39% of the free amino acid pool), and serves as a sulfur and nitrogen source for bacteria, among other functions. A recent study provided experimental evidence of the utilisation of host-derived taurine by sponge symbiont-*Candidatus* Taurinisymbion ianthellae, and of its role in assimilation and energy conservation (Moeller et al. 2023). Moreover, since taurine might be produced by sponges from sulfide, metabolisation of taurine by strain Hal317^T^ might contribute to detoxification processes in the sponge environment.

Urea could be taken up by an ABC transporter (UrtABCDE) and metabolised by the UreABC system to ammonia as revealed in the Hal317^T^ genome (Fig. 4). Urea utilisation, a common trait of sponge-associated microorganisms, further supports the nitrogen needs of bacteria (O'Brien et al. 2024).

Inorganic and organic phosphorus compounds have been reported to be taken up by sponges and their symbionts (Jonas and Hill 2022). Phosphate could be transported into Hal317^T^ cell through both low-affinity phosphate inorganic transport (Pit) and high-affinity phosphate-specific transport (Pst) systems (Surin et al. 1985) (Fig. 4). The genome contains the genes coding for the four components of the Pst system, i.e., PstA, Pstb, PstC, and phosphate binding protein PstS (Martin and Liras 2021). PhoU might be involved in regulation processes in dependency of the phosphate concentration (Martin and Liras 2021). Phosphonate from the dissolved organic phosphate pool is an additional important phosphorus source being available through a substrate-specific pathway for the strain Hal317^T^. Genes coding for the phosphonate ABC transporter, PhnCED were observed. 2-aminoethylphosphonate (AEP) is the most abundant phosphonate in the marine environment, which could be metabolised under the function of phosphonoacetate hydrolase PhnA (Lockwood et al. 2022).

Similar to other sponge symbiont microbes (Engelberts et al. 2020), Hal317^T^ possess part of the pathway for synthesising vitamin B12 (cobalamin), vitamin B5 (pantothenic acid), and vitamin B6 (pyridoxin).

Furthermore, the genome of strain Hal317^T^ contains genes coding for features dealing with different types of stress conditions in the sponge environment (Fig. 4). The pathway of ectoine synthesis was found in Hal317^T^ genome by KEGG and AntiSMASH annotation. As an important osmoprotectants, ectoine might helpful for Hal317^T^ to adapt to saline environments. Survival at fluctuating sodium concentrations and alkaline pH values could be promoted by the presence of cation/proton antiporters, which regulate pH and homeostasis in the cell (Krulwich et al. 2009). Strain Hal317^T^ harbors the Na +/H + antiporter NahA and the Mrp (Mnh) systems coded by the genes *mrpABCDEFG* as well as the K +/H + antiporter NhaP2. Homeostasis could be further maintained by chloride channel proteins (CIC family, YfbK) and magnesium transporters (CorA, MgtE). The cold shock proteins could help Hal317^T^ adapt to temperature shifts. Defense against reactive oxygen species (ROS), such as hydrogen peroxide and superoxide anion causing cellular oxidative damage might be mediated by the superoxide dismutase (SOD), catalase, peroxidases, and DoxX family proteins (Nambi et al. 2015). Such strategy could be important for strain Hal317^T^ because ROS-generating enzymes are widely distributed in sponges (Hewitt and Degnan 2022). Drug/metabolite transporters (DME and RND family), as well as heavy metals exporters (CopA) might endow the strain Hal317^T^ with the ability of extruding antibiotic or other toxic substances in sponge reservoirs. Stress-related denaturation of proteins might be combated by chaperones like the DnaK- and the ClpB-system, which assist the proper protein folding during or after synthesis, and after partial denaturation (Martin et al. 2014). Since excess heme is cytotoxic to cells, strain Hal317^T^ showed a heme exporter system consisting of protein CcmABC.

Modules of toxin-antitoxin (TA) gene families were found in the genome of strain Hal317^T^. Among them were the type II toxin-antitoxin system ParE1/3/4–ParD1/3/4, the toxin families RelE/ParE, PemK/MazF, and VapC as well as the antitoxin families Phd/YefM and HipB. In addition, the type IV toxin-antitoxin system AbiEii–AbiEi was detected. Toxin-antitoxin systems are genetic elements that encode a toxin protein capable of inhibiting cell growth and an antitoxin that counteracts the toxin (Harms et al. 2018). As examples, RelE inhibits the translation during amino acid starvation (Griffin et al. 2013) and bacterial abortive infection (Abi) systems are ‘altruistic’ cell death systems that are activated by phage infection and limit viral replication, thereby providing protection to the bacterial population (Dy et al. 2014; Jahn et al. 2021). In addition, TA-systems such as HipAB mediates antibiotic persistence (Huang et al. 2020) and might support the survival of strain Hal317^T^ to antibacterial compounds produced by the sponge and/or by the microbiome (Sizikov et al. 2020).

Strain Hal317^T^ exhibited two enzymes which degrade acyl-homoserine lactones (AHL). Acyl-homoserine-lactone acylase (EC:3.5.1.97) catalyses the reaction *N*-acyl-l-homoserine lactone + H_2_O→homoserine lactone + carboxylate. The second enzyme, *N*-acyl homoserine lactone hydrolase (EC:3.1.1.81), catalyses the reaction *N*-acyl-l-homoserine lactone + H_2_O→*N*-acyl-l-homoserine + H +. AHL molecules are involved in quorum sensing mediated processes, e.g., biofilm production, virulence, and sporulation. Quorum sensing inhibitors produced by Hal317^T^ could inhibit the growth and/or the persistence of sponge-associated AHL producing bacteria (Grandclément et al. 2016; Britstein et al. 2018) and might also affect gene expression of the sponge host (Schmittmann et al. 2020).

### Unique genomic features of Hal317^T^ compared to type strains of the family *Iamiaceae*

Hal317^T^ shows unique genes or pathways not present in other type strains in the family *Iamiaceae* (Table 3, Supplement Table S1). As mentioned before, phosphonate ABC transporter protein PhnCED, phosphonoacetate hydrolase PhnA and transcriptional regulator PhoB were only shown in the genome of Hal317^T^. In contrast to other members of *Iamiaceae*, strain Hal317^T^ contained the glutamate-uptake system consisting of ABC transporter protein GluABC. Additionally, the non-phosphorylated l-fucose pathway was present in the genome of Hal317^T^. The strain Hal317^T^ possesses a variety of extracellular enzymes that are not found in the genomes of other strains, further highlighting the importance of peptidase and amino acid metabolism to this sponge-derived strain.

### Morphology, physiology, and chemotaxonomy

**Fig. 5:**
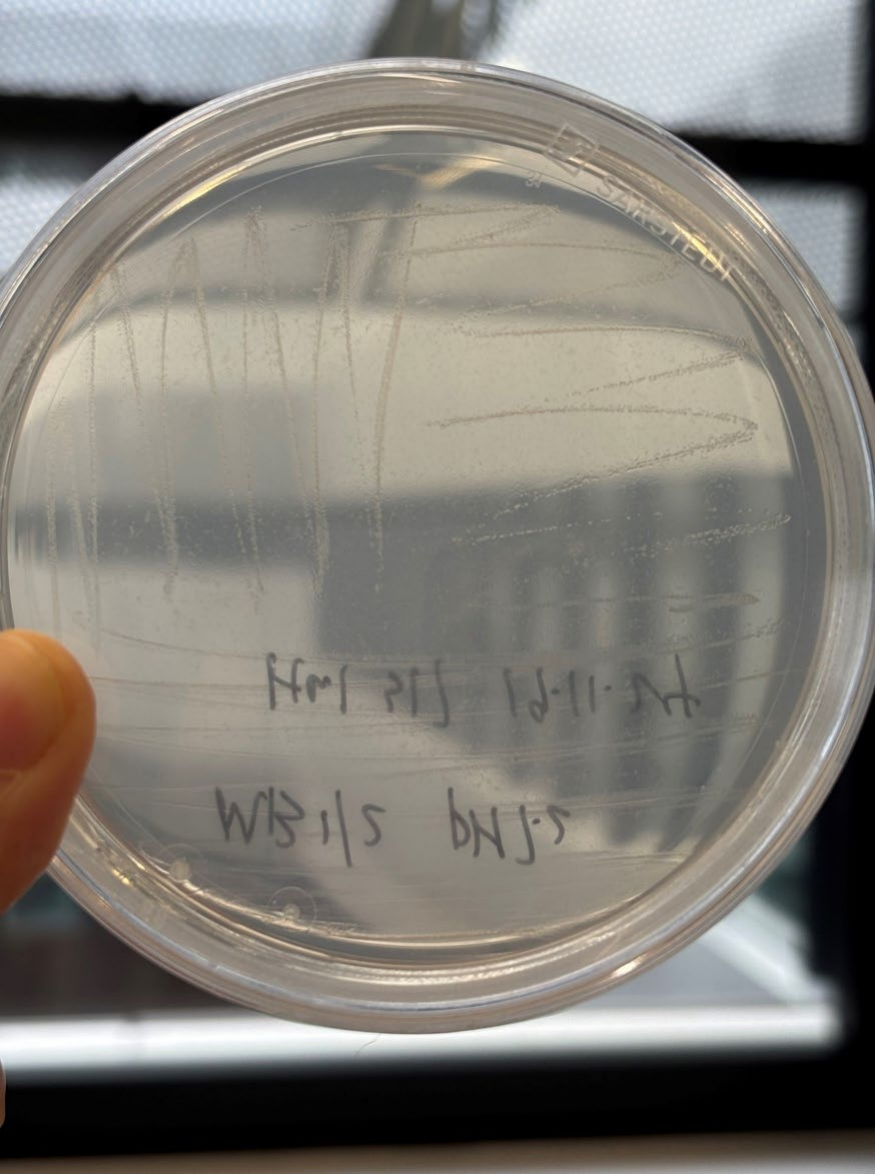
Colony morphology of strain Hal317^T^ after cultivation on half-concentrated MB medium for 7 days at 28 °C

**Fig. 6:**
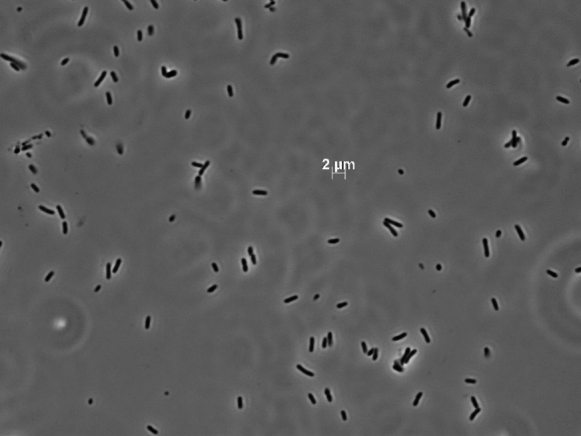
Micrograph of strain Hal317^T^ after cultivation on half-concentrated MB medium for 7 days at 28 °C

Morphological, physiological, and chemotaxonomic features of strain Hal317^T^ in comparison to other type strains of the family *Iamiaceae* are shown in Table 4. Colonies are transparent and 1 mm in diameter (Fig. 5). Cells of strain Hal317^T^ are Gram-positive, non-motile stabs, 0.5 µm wide and 1.5–2.0 µm long (Fig. 6). Growth occurs on 1–2% (w/v) sea salt, with an optimum at 1%. No growth was observed with NaCl as the sole salt supplement. In terms of temperature adaptability, strain Hal317^T^ can tolerate temperatures as low as 5 °C. The strain grew at 5–35 °C with an optimum between 30 and 35 °C and at pH 6.0–8.5 with an optimum pH 6.0–7.0. Besides, the strain Hal317^T^ could survive under microaerophilic circumstances.

**Table 4:** Phenotypic characteristics of strain Hal317^T^ and phylogenetically related type strains in the family *Iamiaceae*

Characteristic	1	2	3	4	5	6	7
Origin	Sponge	Human skin (keratinocytes)	Daechung Reservoir water sample	South China Sea sediment	Saline lake sediment	Hot spring sediment	The abdominal epidermis of a sea cucumber, *Holothuria edulis*
Cell morphology	Rod	Rod	Short rod	Short rod	Short rod	Short rod	Rod
Cell length (μm)	1.5–2	0.8–1.4	0.7–1.0	1.0–2.0	1.1	0.8–1.0	1.2–1.7
Cell width (μm)	0.5	0.4–0.7	0.3–0.5	0.3–0.6	0.5	0.5–0.6	0.3–0.5
Temperature (°C) range (optimum)	5–35 (30–35)	18–37 (25)	15–37 (30)	10–45 (35–37)	28–37 (37)	37–55 (45)	15–40 (28–30)
Salt tolerance (% w/v) range	1–2 (tropic marine salt)	0–1.0 (NaCl)	0–0.5 (NaCl)	0–5.0 (NaCl)	0–6.0 (NaCl)	0–3.0 (NaCl)	0–12.0 (NaCl)
pH range (optimum)	6.0–8.5 (6.0–7.0)	6.0–8.0 (7.0)	7.0 (7.0)	6.0–9.0 (7.0)	6.0–10.0 (8.0)	7.0–8.0 (7.0)	6.0–9.0 (7.0)
Major menaquinones	MK-9(H8)	MK-9(H8)	MK-9(H6)	MK-9(H8)	MK-9(H8)	MK-9(H8)	MK-9(H6)
Diaminopimelic acid	meso-DAP	meso-DAP	NA	dd-DAP	meso-DAP	meso-DAP	meso-DAP
Polar lipids	DPG, GPL, GL, PL	DPG, APL APGL, PGL, L1, L2	DPG, PI, PIM	DPG, PI, PC, PIM	DPG, PI, PIM	DPG, PI, PIM	NA
Major fatty acids	C_18:1_ ω7c, C_16:1_ ω7c, C_17:1_ ω8c	C_16:0_, C_18:1_ ω9c	C_16:1_ ω5c, C_16:0_, C_17:1_ ω8c, C_18:1_ ω9c	C_17:1_ ω8c, C_17:0_	iso-C_16:0_, C_17:0_, C_16:0_	C_16:0_, C_17:0_, C_18:0_, iso-C_16:0_	C_17:0_, C_17:1_ ω8c, C_15:0_, C_16:0_

Strains: 1, Hal317^T^ (CP107030, this study); 2, *Actinomarinicola tropica* SCSIO 58843^T^ (GCA_009650215.1, He et al. 2020); 3, *Aquihabitans daechungensis* CH22-21^T^ (CP172265, Jin et al. 2013); 4, *Dermatobacter hominis* Kera-3^T^ (GCA_020715685.1, Jo et al. 2023); 5, *Iamia majanohamensis* F12^T^ (GCA_028532485.1, Kurahashi et al. 2009); 6, *Rhabdothermincola salaria* EGI L10124^T^ (GCA_021246445.1, Gao et al. 2022); 7, *Rhabdothermincola sediminis* SYSU G02662^T^ (GCA_014805525.1, Liu et al. 2019); APGL, aminophosphoglycolipids; APL, aminophospholipids; DPG, diphosphatidylglycerol; GL, glycolipid; GPL, glycophosholipid; L, lipid; PC, phospatidylcholine; PGL, phosphoglycolipids; PI, phosphatidylinositol; PIM, phosphatidylinositol mannoside; PL, phospholipid; NA, not specified

Strain Hal317^T^ was positive for oxidase, esterase (C4), esterase lipase (C8), leucine arylamidase, valine arylamidase, and cystine arylamidase, weak positive for alkaline phosphatase, α-glucosidase, catalase, lipase (C14), acid phosphatase, naphthol-AS-BI-phosphohydrolase, *β*-galactosidase, *β*-glucosidase, *N*-acetyl-*β*-glucosaminidase, and *α*-fucosidase, negative for trypsin, *α*-chymotrypsin, *α*-mannosidase, *α*-galactosidase, and *β*-glucuronidase.

The major fatty acids identified were C_18:1_ *ω*7*c* (42.3%), C_16:1_ *ω*7*c* (23.1%), C_17:1_ *ω*8*c* (8.4%), and C_16:1_ (7.6%). C_16:1_* ω*5*c* (4.5%), C_18:1_ *ω*9*c* (4.1%), C_18:0_ (2.2%), iso-C_16:0_ (1.4%), C_17:0_ (1.1%), C_14:0_ (0.9%), iso-C_16:1_ *ω*6*c* (0.4%), C_15:1_ *ω*6*c* (0.3%), and C_18:1_ *ω*5*c* (0.3%) were present in minor amounts. In contrast to the other type strains, the major fatty acids C_18:1_ *ω*7*c* and C_16:1_ *ω*7*c* were only present in strain Hal317^T^.

Whole-cell sugar analysis revealed rhamnose, ribose, mannose, and galactose. The major respiratory menaquinone was MK-9(H_8_) with minor amounts of MK-9(H_6_) and MK-9(H_10_). *Meso*-diaminopimelic acid was observed in the whole-cell hydrolysate, which is identical to the overall diaminopimelic acid pattern for the family *Iamiaceae* except *Actinomarinicola tropica* SCSIO 58843^T^.

The polar lipids diphosphatidylglycerol, a glycophospholipid, two glycolipids, four phospholipids, and an unknown lipid were shown for strain Hal317^T^ (Supplement Figure S1).

## Conclusion

*Acidimicrobiia* isolation has so far been notoriously difficult and frequently unsuccessful. In the present study, we report on the isolation of a novel *Acidimicrobiia* strain Hal317^T^ from the Baltic Sea sponge *Halichondria panicea*. Phylogenetic, genomic (ANI < 71.49%, dDDH < 70%, and POCP < 50.30% in contrast to the other type strains from *Iamiaceae*), chemotaxonomic, and physiological features, provided strong evidence, that strain Hal317^T^ represents a member of a novel genus of the family *Iamiaceae*, for which the name *Actinospongicola halichondriae* is proposed. The genome of strain Hal317^T^ exhibited characteristics in adaptation to sponge environments, such as utilisation of diverse carbohydrates present in sponges and synthesis of vitamins. Moreover, genes involved in osmoprotectant synthesis, defense mechanisms against ROS and toxin-antitoxin systems, all contribute to enhancing the stress adaptability of the cell. Strain Hal317^T^ displayed further unique genome features compared to the other type strains in the family *Iamiaceae*, such as a non-phosphorylated l-fucose pathway, a substrate-specific phosphonate pathway, and a glutamate-uptake system.

Our study expands the number of cultured representatives within the class *Acidimicrobiia* and provides new insight into isolating sponge associated microbes. With the presented results, it should be possible to isolate further acidimicrobiial taxa from a variety of sponges, even from the deep sea*.* The combination of natural seawater based low nutrient media supplemented with *N*-acetyl-d-glucosamine, other sponge-derived carbohydrates, or taurine under microaerophilic conditions is a promising approach to culture more of the ever so often cryptic sponge microbiome.

### Description of *Actinospongicola* gen. nov.

*Actinospongicola* (ac.ti´no. Gr. fem. n. *aktis (gen. aktînos)*, a ray, beam; spon.gi´co.la. L. fem. n. *spongia*, a sponge; L. masc./fem. n. suff. *-cola*, inhabitant; from L. masc./fem. n. *incola*, dweller; N.L. masc./fem. n. *spongicola*, inhabitant of sponges).

Cells are Gram-positive, rod-shaped, microaerophilic, and non-motile. The major fatty acids are C_18:1_ *ω*7*c*, C_16:1_ *ω*7*c*, C_17:1_ *ω*8, and C_16:0_. Major respiratory menaquinone is MK-9(H_8_). The major polar lipids are diphosphatidylglycerol, glycophospholipid, glycolipids, and phospholipids.

### Description of Actinospongicola halichondriae sp. nov.

*Actinospongicola halichondriae* (ha.li.chon'dri.ae. N.L. gen. n. *halichondriae*, of the sponge genus *Halichondria*).

Cells are 0.5 µm wide and 1.5–2 µm long. Colonies are transparent and 1 mm in diameter. Growth occurs on 1–2% (w/v) sea salt (optimum 1%), at 5–35 °C (optimum 30–35 °C), and at pH 6.0–8.5 (optimum pH 6.0–7.0). Strain is positive for oxidase, catalase, esterase (C4), esterase lipase (C8), leucine arylamidase, valine arylamidase, cystine arylamidase, alkaline phosphatase, *α*-glucosidase, lipase (C14), acid phosphatase, naphthol-AS-BI-phosphohydrolase, *β*-galactosidase, *β*-glucosidase, and *N*-acetyl-*β*-glucosaminidase, but negative for trypsin, *α*-chymotrypsin, *α*-mannosidase, *α*-galactosidase, and *β*-glucuronidase. Cell wall sugars are rhamnose, ribose, mannose, galactose, and *meso*-diaminopimelic acid. The DNA G + C content of Hal317^T^ is 68.70%.

The type strain Hal317^T^ (= DSM 114536^T^ = LMG 32795^T^) was isolated from the marine sponge *Halichondria panicea* collected at Schilksee along the Kiel-Fjord of the Baltic Sea (latitude 54.424705, longitude 10.175133).

## Supplementary Information

Below is the link to the electronic supplementary material.

Supplementary file1 (DOCX 787 KB)

Supplementary file2 (XLSX 133 KB)

## Data Availability

Sequence data that support the findings of this study have been deposited as follows. The GenBank accession number for the 16S rRNA gene sequence of strain Hal317T is MT406698. Bioproject and Biosample accession numbers of strain Hal317T are PRJNA1144641 and SAMN42567621, respectively. The GenBank accession number for the genome sequence of strain Hal317T is CP171622. Bioproject and Biosample accession numbers of strain Aquihabitans daechungensis DSM 27986 T are PRJNA1160487 and SAMN43755886, respectively. The accession number of the genome sequence of Aquihabitans daechungensis DSM 27986 T is CP172265. Polar lipids profile of strain Hal317T is provided within the supplementary information file.

## Abbreviations

BCCM/LMG: Belgian coordinated collections of microorganisms
bp: Base pairs
BSW: Baltic Sea water
DSMZ: Leibniz-Institut DSMZ-Deutsche Sammlung von Mikroorganismen und Zellkulturen
MB: Marine medium
rpm: Revolutions per minute
