# Supplementary material for: Genomic characterisation of Actinospongicola halichondriae gen. nov., sp. nov., the first sponge-derived cultivated representative of a new genus within the class Acidimicrobiia
Source: Antonie Van Leeuwenhoek. 2025 Jul 15;118(8):113. doi: 10.1007/s10482-025-02126-4 (PMC12263773; doi:10.1007/s10482-025-02126-4)
Supplement: Supplementary file 1 — Supplementary file1 (DOCX 787 KB) [file 10482_2025_2126_MOESM1_ESM.docx]

**Supplement**

**Genomic characterisation of *Actinospongicola halichondriae* gen. nov. sp. nov., the first sponge-derived cultivated representative of a new genus within the class *Acidimicrobiia***

Jing Huang, Jutta Wiese, Leon X. Steiner, Tanja Rahn, Erik Borchert, Ute Hentschel

**Figure S1** Polar lipids profile of strain Hal317^T^


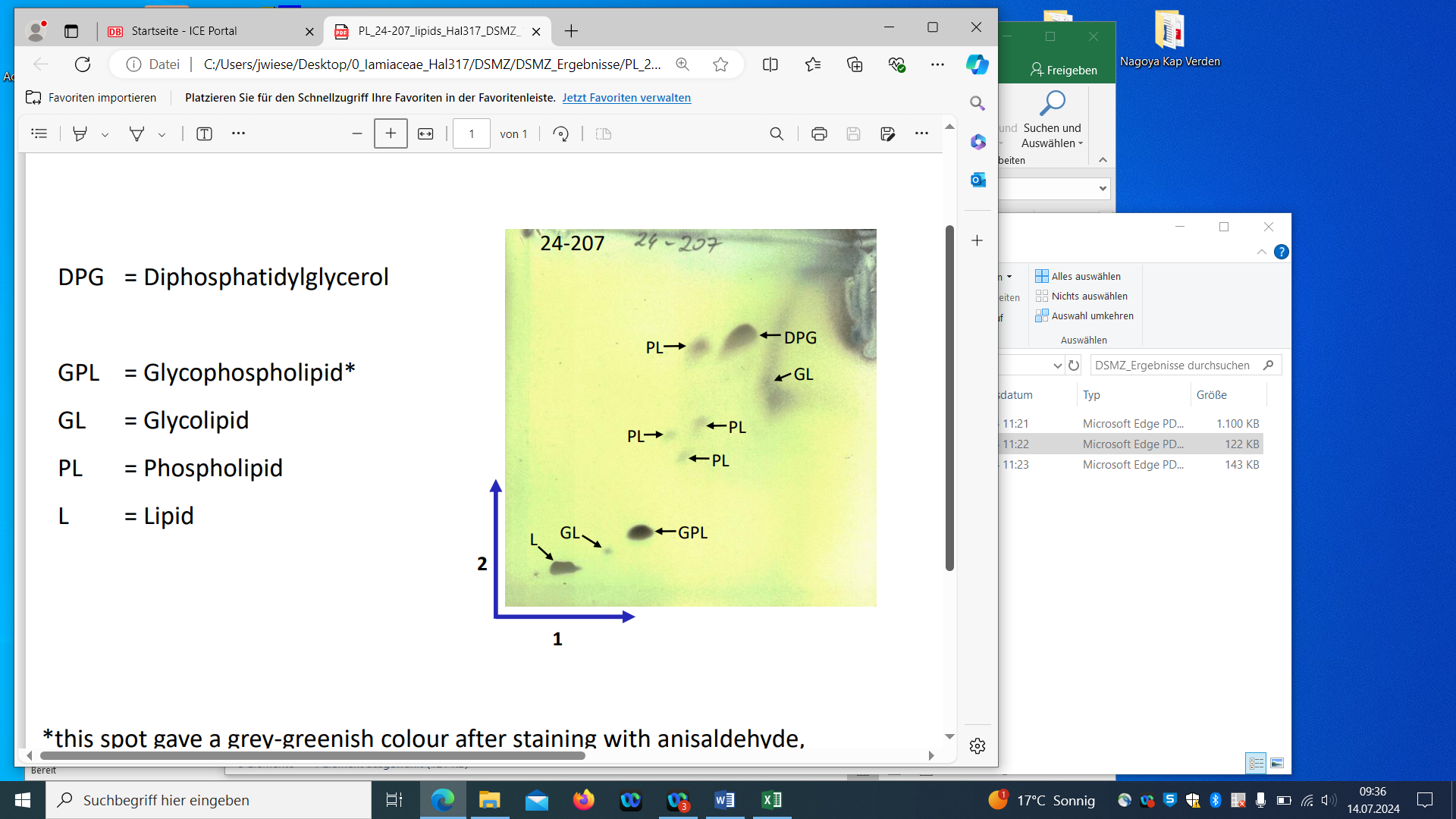


Legend: DPG, diphosphatidylglycerol; GL, glycolipid; GPL, glycophosholipid; L, lipid; PL, phospholipid
